# Estudio de las variantes alélicas CYP2C9*2 y CYP2C9*3 en muestras de población mestiza peruana

**DOI:** 10.7705/biomedica.4636

**Published:** 2019-09-01

**Authors:** Ángel Tito Alvarado, Ana María Muñoz, Berta Loja, Jessica Michiko Miyasato, Jorge Antonio García, Roberto Andrés Cerro, Luis Abel Quiñones, Nelson Miguel Varela

**Affiliations:** 1 Departamento de Biología y Química, Facultad de Ingeniería, Universidad San Ignacio de Loyola (USIL), Lima, Perú Universidad San Ignacio de Loyola Departamento de Biología y Química Facultad de Ingeniería Universidad San Ignacio de Loyola Lima Peru; 2 Clínica Médica Master Medic ERS, Lima, Perú Clínica Médica Master Medic ERS Lima Perú; 3 Facultad de Farmacia y Bioquímica, Universidad Nacional San Luis Gonzaga, Ica, Perú Universidad Nacional San Luis Gonzaga Facultad de Farmacia y Bioquímica Universidad Nacional San Luis Gonzaga Ica Peru; 4 Laboratorio de Carcinogénesis Química y Farmacogenética, Departamento de Oncología Básico-Clínico, Facultad de Medicina, Universidad de Chile, Santiago, Chile Universidad de Chile Departamento de Oncología Básico-Clínico Facultad de Medicina Universidad de Chile Santiago Chile

**Keywords:** citocromo P-450 CYP2C9, genotipo, farmacogenética, Perú, Cytochrome P-450 CYP2C9, genotype, pharmacogenetics, Perú

## Abstract

**Introducción.:**

El citocromo CYP2C9 metaboliza, aproximadamente, el 15 % de los fármacos prescritos. Su gen presenta alelos cuyas frecuencias difieren entre grupos étnicos y poblaciones. Los alelos CYP2C9*2 y CYP2C9*3 dan cuenta de una enzima con actividad disminuida cuya frecuencia no ha sido determinada en la población mestiza peruana.

**Objetivo.:**

Caracterizar la frecuencia de las variantes *2 (rs1799853) y *3 (rs1057910) del gen *CYP2C9* en muestras de población mestiza peruana provenientes de Lima, Tacna y Junín.

**Materiales y métodos.:**

Se hizo un estudio descriptivo, observacional y prospectivo, con muestreo no probabilístico, por conveniencia e incidental. Se incluyeron 218 sujetos según los criterios de inclusión y exclusión; todos los participantes otorgaron su consentimiento informado. El ADN genómico se obtuvo mediante hisopado de mucosa oral, y la detección de los genotipos para los alelos CYP2C9*2 y CYP2C9*3 se hizo mediante reacción en cadena de la polimerasa (PCR) en tiempo real, utilizando sondas TaqMan™.

**Resultados.:**

Las variantes de CYP2C9*2 y CYP2C9*3 están presentes en la población mestiza peruana con frecuencias de 0,046 y 0,062, respectivamente. El análisis de las frecuencias genotípicas observadas permitió predecir que la frecuencia de fenotipos metabolismo intermedio sería del 15,13 % (CYP2C9*1/*2: 5,96 %; CYP2C9*1/*3: 9,17 %), y la de fenotipos de metabolismo lento, del 3,22 % (CYP2C9*2/*2: 1,38 %; CYP2C9*3/*3: 1,38 %; CYP2C9*2/*3: 0,46 %).

**Conclusiones.:**

Se lograron determinar las frecuencias genotípicas y alélicas para las variantes *2 y *3 del gen *CYP2C9* en una muestra no probabilística de población mestiza peruana. Las frecuencias obtenidas (0,046 y 0,062, respectivamente) están entre las esperadas para una población mestiza sudamericana con ascendencia amerindia, europea, africana y asiática.

## Abstract (en)

**Introduction:**

CYP2C9 metabolizes approximately 15% of the prescribed drugs. Its gene has alleles whose frequencies differ between ethnic groups and populations. The alleles CYP2C9*2 and CYP2C9*3 account for an enzyme with decreased activity and their frequencies have not been determined in the Peruvian mestizo population.

**Objective:**

To characterize the frequencies of the allelic variants *2 (rs1799853) and *3 (rs1057910) of *CYP2C9* gen in the Peruvian mestizo population from Lima, Tacna y Junín.

**Materials and methods:**

We conducted an observational, prospective cross-sectional study with non-probabilistic, by convenience, and incidental sampling. We included 218 subjects according to the inclusion and exclusion criteria, all of whom had signed the informed consent. We obtained the genomic DNA from oral mucosa swab. For the detection of the CYP2C9*2 and CYP2C9*3 genotypes, we used real-time-polymerase chain reaction with TaqMan® probes.

**Results:**

The genotyping revealed that CYP2C9*2 and CYP2C9*3 variants have low frequencies (0.046 and 0.062, respectively). The frequency of intermediate metabolizers was 15.13% (CYP2C9*1/*2: 5.96%; CYP2C9*1/*3: 9.17%) and that of slow metabolizers was 3.22% (CYP2C9*2/*2: 1.38%; CYP2C9*3/*3: 1.38%; CYP2C9*2/*3: 0.46%).

**Conclusions:**

It was possible to determine the genotypic and allelic frequencies for the variants *2 and *3 of the *CYP2C9* gene in a non-probabilistic sample of the Peruvian mestizo population. The frequencies obtained (0.046 and 0.062, respectively) corresponded to those expected for a South American mestizo population with Amerindian, European, African and Asian ancestry.

En la práctica clínica se sabe que los fármacos son eficaces en 25 a 60 % de los pacientes, es decir que la cantidad de quienes presentan falla terapéutica es considerable ^1^. Este fenómeno responde a múltiples factores, entre los cuales la farmacocinética clínica y la genética explican del 25 al 95 % de los casos, en los que, frente a una misma dosis, se registran concentraciones plasmáticas por debajo de la concentración mínima efectiva (CME) o por encima de la concentración máxima efectiva (CME), asociadas con una menor eficacia de los fármacos o con efectos adversos de diversa consideración. Esta situación se hace especialmente relevante en fármacos de estrecho margen terapéutico ^1^^-^^3^.

En este contexto, la medicina personalizada, consistente en adaptar el tratamiento farmacológico de determinadas enfermedades a la dosis correcta en el momento adecuado con base en las características de cada paciente, su sexo, etnia (o mestizaje), estilo de vida y perfil genético-molecular, así como al nivel del fármaco en el estado estacionario, minimiza los efectos secundarios y optimiza la eficacia farmacológica ^2^^,^^3^.

Los genes que codifican aquellas enzimas que metabolizan la mayoría de los fármacos presentan polimorfismos genéticos, incluidos la ausencia completa del gen, los de un solo nucleótido (*single nucleotide polymorphism*, SNP) y las duplicaciones génicas, cuyos efectos van desde la ausencia en la expresión de la enzima hasta la reducción, alteración o incremento de su actividad.

El gen *CYP2C9,* situado en el brazo largo del cromosoma 10 en la región 24 (10q24) ^4^, presenta alelos cuyas frecuencias difieren entre los grupos étnicos y dependen de la ubicación geográfica de una determinada población ^5^. La enzima CYP2C9 (miembro del sistema oxidasa de función mixta, citocromo P450: EC 1.14.13.48) metaboliza, aproximadamente, el 15 % de los fármacos ^1^ y el gen que la codifica presenta más de 60 variantes genéticas ^6^ del tipo SNP ^5^, algunas de las cuales forman parte de las seis variantes alélicas de importancia clínica.

El alelo CYP2C9*1 da cuenta de un metabolismo normal ^7^, con una actividad enzimática del 100 % ^8^^,^^9^. Los alelos implicados en la disminución del metabolismo son el CYP2C9*2 y el CYP2C9*3, cuyas frecuencias difieren entre grupos étnicos ^9^^,^^10^, siendo más altas en los caucásicos que en los africanos y asiáticos ^5^.

La variante alélica CYP2C9*2 (rs1799853: g.3608C>T, c.430C>T, exón 3; cambio en la proteína de Arg144Cys) se asocia con una menor interacción de la enzima con su cofactor, reduciendo la velocidad máxima (Vmáx) de 12 % ^2^^,^^4^^,^^8^ a 50 % con respecto a la enzima de actividad normal codificada por el alelo CYP2C9*1^11^.

El alelo CYP2C9*3 (rs1057910: exón 7, g.42614A>C, c.1075A>C; cambio en la proteína de Ile359Leu) tiene una actividad del 5 % debido a una variación en el sitio de unión al sustrato ^3^, lo que genera un aumento de la constante de Michaelis-Menten (Km) y una reducción en la Vmáx ^11^.

Cuando un individuo porta dos alelos de función reducida, sean estos CYP2C9*2/*2, CYP2C9*3/*3 o CYP2C9*2/*3, se configura un genotipo predictor de metabolismo lento ^4^^,^^9^. Los genotipos implicados en el metabolismo intermedio se generan cuando se presenta un alelo de función normal y otro de función reducida (CYP2C9*1/*2 ó CYP2C9*1/*3) ^4^^,^^9^.

El gen *CYP2C9* está implicado en el metabolismo de medicamentos anticonvulsivos, anticoagulantes, antihipertensivos, antiinflamatorios no esteroides e hipoglucemiantes, y se ha demostrado que la presencia de genotipos predictores de metabolismo lento en el gen *CYP2C9* se asocia con una mayor frecuencia de efectos secundarios de varios de estos fármacos.

La fenitoína, anticonvulsivo ampliamente utilizado en el tratamiento de la epilepsia, es metabolizada en más del 90 % por el *CYP2C9*, y la presencia de determinados genotipos del gen condiciona sus parámetros farmacocinéticos, aumentando o disminuyendo su metabolismo ^7^. En los caucásicos se ha reportado que los genotipos CYP2C9*1/*2, CYP2C9*1/*3^12^^,^^13^ y CYP2C9*2/*2 son responsables de los efectos secundarios de la fenitoína ^12^^-^^14^; en los individuos indios, el CYP2C9*3/*3^12^^,^^15^, en japoneses, los genotipos CYP2C9*1/*3 y CYP2C19*1/*3^12^, y en los afroamericanos están implicados los genotipos CYP2C9*6/*6 y CYP2C19*1/*1^12^. Además, el *CYP2C9* metaboliza el clopidogrel ^16^^,^^17^, la S-warfarina ^11^, la fluvastatina ^6^, la glibenclamida, el losartán, el irbesartán, el ibuprofeno, el lornoxicam y el diclofenaco ^18^.

En estudios sobre el perfil genético de la población mestiza peruana se ha evidenciado que entre el 68,3 % ^19^ y el 76 % ^20^ es amerindio; entre el 26 ^19^^)^ y el 29 % ^21^, europeo; entre el 2 ^20^ y el 3,2 % ^19^, africano, y el 2,5 % ^19^, asiático; esto último debido especialmente a las migraciones japonesa y china.

Al revisar la base de datos de PubMed-NCBI de estudios de variantes genéticas en enzimas implicadas en el metabolismo de los fármacos, se aprecia la ausencia de trabajos en la población peruana, situación que incluye el estudio del *CYP2C9.* Los estudios de asociación genotípica y fenotípica de ciertos alelos del *CYP2C9* han revelado la importancia de conocer y describir las frecuencias de los alelos del gen *CYP2C9*, dada su relevancia para el metabolismo de varios fármacos de estrecho rango terapéutico y de uso extendido en salud pública, como los anticonvulsivos, ampliamente utilizados en el Perú, en donde la prevalencia de la epilepsia en una población de 31'151.647 fluctúa entre el 0,6 (186.910 pacientes) y el 1,8 % (560.730 pacientes) ^22^.

En este contexto, en el presente estudio se propuso caracterizar las frecuencias de los alelos CYP2C9*2 y CYP2C9*3 -los de mayores frecuencias alélicas globales del gen *CYP2C9*- ^23^ en muestras de población mestiza peruana y compararlas con los datos previamente reportados en otras poblaciones, españolas, africanas y asiáticas.

## Materiales y métodos

Tipo y diseño del estudio

Se llevó a cabo un estudio descriptivo, observacional y prospectivo en el Laboratorio de Biología de la Universidad San Ignacio de Loyola de Lima, Perú. Las personas fueron convocadas e informadas sobre el estudio entre enero y diciembre de 2017.

Tipo de muestreo

Se hizo un muestreo no probabilístico, debido a que las muestras se recogieron en un proceso que no brindaba a todos los individuos de la población iguales oportunidades de ser seleccionados, y por conveniencia e incidental (casual), ya que las muestras se seleccionaron directamente de individuos voluntarios. La población representativa estuvo conformado por residentes de Lima, de la región andina (Junín) y de la costera (Tacna).

Criterios de inclusión y exclusión

Los criterios de inclusión fueron: ser mayor de 18 años, sin relación de parentesco con otros participantes, haber nacido en el Perú, estar radicado en Lima, Junín o Tacna, y ser considerado mestizo por un fenotipo visible, por su región de procedencia (andina o costa) y por tener apellido de ascendencia española, africana, japonesa o china.

En el examen clínico, a cargo de un médico cirujano, los participantes debían presentar un buen estado de salud, presión arterial sistólica de 110- 139 mm Hg y diastólica de 60-89 mm Hg, circunferencia abdominal menor de 95 cm en hombres y de 82 cm en mujeres, no tener diagnóstico de diabetes ni haber estado en tratamiento farmacológico reciente (en los seis meses previos a la fecha de reclutamiento), y no presentar dependencia del alcohol ni de otras drogas de abuso, lo que se estableció mediante una entrevista.

Todos los voluntarios firmaron el consentimiento informado, para lo cual no debían presentar impedimentos físicos ni mentales.

Se excluyeron del estudio a todos las personas con, al menos, un apellido asociado a etnias distintas a las indicadas en los criterios de inclusión.

Obtención del ADN genómico

El ADN genómico se obtuvo mediante hisopado bucal de tejido epitelial plano estratificado no queratinizado. El interior de la mejilla se frotó cinco a seis veces con el hisopo para garantizar una cantidad adecuada de células descamativas. Posteriormente, el hisopo se sumergió durante 60 segundos en 300 μl de solución tampón de lisis y la mezcla resultante se refrigeró a 4 °C por un tiempo no superior a 18 horas.

El ADN genómico (ADNg) se extrajo utilizando el innuPRE DNA Master kit™ (Analytik Jena™), según el protocolo del fabricante, procedimiento realizado en el Laboratorio de Biología de la Universidad San Ignacio de Loyola. El ADN genómico se cuantificó por espectrofotometría utilizando el equipo Denovix (modelo DS-11, FX, Serie Spectrophotometer™, USA).

Se consideraron como muestras aptas para ser sometidas a análisis aquellas con razones de absorbancias de 260/280 nm y 260/230 nm iguales o superiores a 1,8. Las muestras se conservaron a -20 °C hasta su análisis.

Análisis genotípico

Los genotipos se determinaron mediante PCR en tiempo real (RT-PCR) y la identificación de las variantes alélicas, mediante sondas TaqMan para ensayos de genotipificación (Thermo Fischer Scientific Inc., número de catálogo: 4362691) capaces de discriminar los SNP identificados como rs1799853 y rs1057910 para CYP2C9*2 y CYP2C9*3, respectivamente.

El alelo CYP2C9*1 no se determinó directamente; todos los alelos que resultaron negativos para los SNP rs1799853 y rs1057910 se designaron como CYP2C9*1, lo cual es aceptable cuando las variaciones de secuencia correspondientes a alelos no se determinan, por lo que no es erróneo asumir que No * X = *1 ^24^.

En el protocolo estándar, la mezcla de reacción fue de 20 ng de ADN genómico, 5 μl de la 2X Genotyping Master Mix™ (N° de catálogo 4371355), 0,5 μl del 20X TaqMan SNP Genotyping Assay™ (con las sondas e iniciadores correspondientes) (Thermo Fisher Scientific Inc.) y agua de grado biología molecular libre de nucleasas (HyPure,HyClone™) en cantidad suficiente para 10 μl de volumen final de reacción. Para determinar el rs1799853, se utilizó el TaqMan SNP Genotyping Assay™, ID: C 25625805_10, que discrimina la transición NG_008385.1:g.8633C>T, en la secuencia contexto GATGGGGAAGAGGACATTGAGGAC[C/T] GTGTTCAAGAGGAAGCCCGCTGCCT; y para el rs1057910, se utilizó el ensayo ID: C 27104892_10, que discrimina la transversión NG_008385.1:g.47639A>C en la secuencia contexto TGTGGTGCACGAGGTCCAGAGATAC[C/A] TTGACCTTCTCCCCACCAGCCTGCC.

Para la amplificación, se utilizó el equipo Stratagene Mx3000P™ (Agilent Technologies, Waldbronn, Alemania); el programa incluyó un ciclo inicial de desnaturalización a 95 °C durante 10 minutos y 50 ciclos de 15 segundos de desnaturalización a 92 °C, 90 segundos de alineamiento a 60 °C y 60 segundos de elongación a 72 °C.

Esta etapa del proceso experimental se llevó a cabo en el Laboratorio de Carcinogénesis Química y Farmacogenética de la Facultad de Medicina de la Universidad de Chile.

Análisis estadístico

Los análisis estadísticos se hicieron con el programa estadístico informático SPSS™ de IBM. Para determinar si la distribución de los genotipos estudiados seguía el modelo de Hardy-Weinberg, se utilizó la prueba de bondad de ajuste de ji al cuadrado, considerando dos grados de libertad y un valor de alfa menor de 0,05. Los valores de ji al cuadrado mayores de 5,99 en la comparación indicaban el rechazo de la hipótesis nula, por lo tanto, las frecuencias observadas difirieron significativamente de las esperadas ^25^.

Consideraciones éticas

Los sujetos firmaron el procedimiento de consentimiento informado de forma individual y aceptaron su participación voluntariamente después de escuchar las explicaciones sobre los objetivos, riesgos, importancia y aplicación del estudio.

El estudio se ajustó estrictamente a las normas éticas de la Declaración de Helsinki ^26^ y a la reglamentación nacional. Asimismo, fue aprobado por el Comité Institucional de Ética en Investigación de la Universidad de San Martín de Porres mediante oficio N° 582-2017-CIEI-USMP-CCM.

## Resultados

En el cuadro 1 se presentan las características antropométricas de los voluntarios seleccionados (218 peruanos mestizos: 158 hombres y 60 mujeres), señalando su procedencia geográfica. En el cuadro 2 se presentan las frecuencias genotípicas y alélicas observadas para las variantes *2 (rs1799853) y *3 (rs1057910) del gen *CYP2C9*. En el cuadro 3 se presentan por separado las frecuencias genotípicas y alélicas de las muestras de las poblaciones de Lima, Tacna y Junín.

**Cuadro 1:** Procedencia geográfica y características demográficas de la muestra de peruanos mestizos incluida en el estudio

Procedencia	n (%)
Lima	118 (54,12)
Junín	50 (22,94)
Tacna	50 (22,94)
Total	218 (100,00)
**Características demográficas**	
Sexo	
Mujeres	158 (72,48)
Hombres	60 (27,52)
Otras	Promedio ± DE
Edad (años)	24,99 ± 4,75
Peso (kg)	75,43 ± 5,73
Talla (m)	1,67 ± 0,09
IMC (kg/m^2^)	27,16 ± 3,30

DE: desviación estándar

**Cuadro 2:** Frecuencias génicas y genotípicas para los alelos *2 y *3 del gen

	Genotipo	n	%	Alelo	n	frecuencia
CYP2C9*2	C/C (*1/*1)	201	92,20	T (*2)	20	0,046
rs1799853	C/T (*1/*2)	14	6,42			
	T/T (*2/*2)	3	1,38	C	416	0,954
	Total	218	100,00			
CYP2C9*3	A/A (*1/*1)	194	88,99	C (*3)	27	0,062
rs1057910	A/C (*1/*3)	21	9,63			
	C/C (*3/*3)	3	1,38	A	409	0,938
	Total	218	100,00			

**Cuadro 3:** Frecuencias genotípicas y alélicas para las variantes *2 y *3 del gen *CYP2C9* en las tres poblaciones mestizas peruanas incluidas en el estudio

Genotipo	n (%)			Alelo	frecuencia		
	Lima	Tacna	Junín		Lima	Tacna	Junín
C/C (*1/*1)	109 (92,3)	45 (90,0)	47 (94,0)	T (*2)	0,042	0,060	0,040
C/T (*1/*2)	8 (6,8)	4 (8,0)	2 (4,0)				
T/T (*2/*2)	1 (0,9)	1 (2,0)	1 (2,0)				
Total	118 (100,0)	50 (100,0)	50 (100,0)				
A/A (*1/*1)	99 (83,9)	48 (96,0)	47 (94,0)	C (*3)	0,093	0,020	0,030
A/C (*1/*3)	16 (13,6)	2 (4,0)	3 (6,0)				
C/C (*3/*3)	3 (2,5)	0 (0,0)	0 (0,0)				
Total	118 (100,0)	50 (100,0)	50 (100,0)				

La prueba de bondad de ajuste de ji al cuadrado para comprobar el equilibrio de Hardy-Weinberg de las frecuencias genotípicas en estudio, arrojó los siguientes valores: 3,197 y 4,624 para la muestra de población proveniente de Lima; 4,228 y 0,021 para la de Tacna, y 11,480 y 0,048 para la de Junín. Considerando que los valores de ji al cuadrado mayores de 5,99 negaban la hipótesis nula, solo las frecuencias observadas para los genotipos del alelo CYP2C9*2 en la muestra de Junín difirieron significativamente de las esperadas (χ2=11,480). En los demás casos, los valores de χ2 obtenidos no permitieron rechazar la hipótesis nula, y se consideró que las muestras estaban en equilibrio de Hardy-Weinberg.

Se reporta la presencia de los genotipos en el gen *CYP2C9* que predicen un metabolismo extenso (CYP2C9*1/*1), uno intermedio (CYP2C9*1/*2, *1/*3) o uno lento (CYP2C9*2/*2, *3/*3 y 2*/3* ) (cuadro 4).

**Cuadro 4:** Predicción de la frecuencia de fenotipos metabólicos en la población mestiza peruana según las frecuencias genotípicas observadas para los alelos *2 y *3 del gen *CYP2C9*

Fenotipos metabólicos	Porcentaje esperado en la población mestiza peruana	Genotipo predictivo	Genotipos observados n (%)
Extensivos (ME)	81,65	*1/*1^a^	178 (81,65)
Intermedios (MI)	15,13	*1/*2^b^	13 (5,96)
		*1/*3^c^	20 (9,17)
Lentos (ML)	3,22	*2/*2	3 (1,38)
		*3/*3	3 (1,38)
		*2/*3^d^	1 (0,46)

a sujetos que no portaban ninguno de los alelos en estudio (*2 y *3)

b sujetos heterocigotos para *2 que no presentaban el alelo *3

c sujetos heterocigotos para *3 que no presentaban el alelo *2

d sujetos que resultaron ser heterocigotos para *2 y *3

## Discusión

En diversos estudios a nivel internacional, se reportan importantes variaciones en las frecuencias de los alelos CYP2C9*2 y CYP2C9*3 en distintas poblaciones o etnias.

En el presente estudio se determinaron las frecuencias genotípicas y alélicas para estas variantes en una muestra de población mestiza del Perú, de las cuales no hay reportes previos. Las frecuencias observadas para el rs1799853 (CYP2C9*2) y el rs1057910 (CYP2C9*3) fueron de 0,046 y 0,062, respectivamente.

Al comparar estos resultados con otras poblaciones, se encontró que la frecuencia para el alelo *2 es similar a la reportada para la población de Bolivia (0,048) ^27^, pero es superada por la de las poblaciones chilena ^28^, brasileña ^29^ y, más ampliamente, por la de la española-caucásica ^30^.

Al analizar por separado la presencia de esta variante (*2) en las muestras de la población de Lima, Tacna y Junín, se observó una similitud entre las frecuencias obtenidas para Lima (0,042) y Junín (0,040), las cuales resultaron ser un 30 % menores a la observada en Tacna (0,060).

Por otro lado, la frecuencia del alelo *3 (0,062) es similar a las reportadas en poblaciones españolas (0,062 - 0,083) ^30^. Sin embargo, hubo una amplia diferencia entre las frecuencias del alelo *3 de la muestra de Lima (0,093) y las bajas frecuencias de las muestras de Tacna (0,020) y Junín (0,030).

Una posible hipótesis explicativa de estas diferencias es la deriva génica sufrida por estas variantes polimórficas en la población mestiza peruana, dada la frecuencia registrada para estos alelos en las etnias originarias ^4^, lo que podría haber favorecido la generación de distintos patrones de mestizaje ^19^^-^^21^.

Las frecuencias obtenidas para las variantes *2 y *3 en la muestra de población peruana superan las reportadas para poblaciones africanas, chinas y japonesas, y son inferiores a las obtenidas en poblaciones españolas o caucásicas, tal como lo señalaron Bravo-Villalta, *et al*. ^27^ al observar que la frecuencia del CYP2C9*2 y CYP2C9*3 es muy baja en los africanos (0,010 y 0,005, respectivamente).

Por su parte, Vicente, *et al*. ^31^, informan que en españoles (n=223) la frecuencia es mayor para ambos alelos (*2: 0,133; *3: 0,077). Estos resultados concuerdan con la revisión de Céspedes-Garro, *et al*. ^4^, quienes señalan que el alelo CYP2C9*2 es el más frecuente en las poblaciones caucásicas (0,140), poco frecuente en los nativos americanos (0,013), e incluso menor en los asiáticos del este (0,006) y en los africanos (0,005), en tanto que el alelo CYP2C9*3 es más frecuente entre los asiáticos del sur (0,117).

En una reciente revisión realizada por Daly, *et al*. ^23^, se plantea que las variantes en cuestión son las de mayor frecuencia para el gen *CYP2C9*, con frecuencias globales de 0,0914 para el alelo *2 y de 0,0637 para el alelo *3. En la misma revisión se detallan las frecuencias en diferentes grupos poblacionales y se señala que los europeos (caucásicos) tienen las frecuencias más altas para el alelo *2 (0,1268) y, los habitantes del sur de Asia, para el alelo *3 (0,1131).

Los genotipos observados para los dos alelos estudiados en las muestras provenientes de Lima, Tacna y Junín guardan el equilibrio de Hardy- Weinberg, excepto por la distribución de los genotipos para el alelo *2 en la muestra de Junín, lo cual se explicaría por factores propios de la población, como la selección por conveniencia, las migraciones, o por las limitaciones propias de este estudio, es decir, el tamaño de la muestra de las poblaciones de Junín y Tacna (n=50) y el método de muestreo utilizado (no probabilístico).

Los dos alelos estudiados están involucrados en la predicción de fenotipos metabólicos intermedios y lentos, lo que es relevante en la práctica clínica asistencial. Cuando se establece que un paciente es portador de uno o ambos alelos (*2, *3 o ambos), se da cuenta de una mayor propensión

a presentar efectos secundarios tras la administración de fármacos metabolizados por la enzima CYP2C9.

Al establecer la frecuencia de estos alelos en una determinada población, es posible sugerir recomendaciones o modificaciones de las guías clínicas o de los protocolos de tratamiento y hacer un mejor análisis e interpretación de los programas de farmacovigilancia ^1^. En este sentido, el presente estudio aspira a que los médicos especialistas y los farmaceutas en los hospitales consideren estas variables genéticas como base para promover la implementación de la medicina de precisión, así como a promover futuras investigaciones en el área de la farmacogenética en Perú.

El estudio constituye la primera determinación de las frecuencias genotípicas y alélicas para las variantes *2 y *3 del gen *CYP2C9* en la población mestiza peruana. Las frecuencias obtenidas (0,046 y 0,062, respectivamente) son las esperadas para una población mestiza suramericana con ascendencia amerindia, europea (esencialmente española), africana y asiática (especialmente por migración japonesa y china). Las frecuencias de estos alelos son similares a las reportadas en países latinoamericanos vecinos como Chile y Bolivia, y en el contexto global, son menores que las de la población europea (caucásica), pero superiores a las informadas para las etnias africanas y asiáticas.
